# Neonatal Lung Diseases: A Clinical Potential for Sex Steroids and a Novel Intracrine Organ

**DOI:** 10.3389/fmed.2021.664969

**Published:** 2021-05-05

**Authors:** Yves Tremblay, Alexia Morin-Labbé

**Affiliations:** ^1^Reproduction Axis, Perinatal and Child Health, CRCHU de Québec, Québec, QC, Canada; ^2^Department of Obstetric, Gynecology & Reproduction, Faculty of Medicine, Laval University, Québec, QC, Canada; ^3^Centre de Recherche en Reproduction, Développement et Santé Intergénérationnelle, Laval University, Québec, QC, Canada

**Keywords:** neonatal, lung, steroid, intracrinology, sex steroid, androgen

## Introduction

### Intracrinology

Intracrinology refers to the production of active sex steroids *in situ* within the cells where the action takes place. The active hormones are not released in the extracellular space (1, 2) as opposed to endocrinology where glands release active hormones in circulation to exert their effect on target tissues. The term “intracrinology” has been coined 30 years ago during the study of the prostate (3). It has been shown that after removing androgens of testicular origin, the prostate still produces dihydrotestosterone (DHT) from an inactive circulating sex steroid precursor of adrenal origin. Can this concept of intracrinology be extended to the developing lung?

### Intracrinology and Lung Development

Mammalian lung development divides into 5 overlapping stages. When the embryonic, pseudo-glandular and canalicular stages end, the formation of terminal bronchioles with terminal sacs begins. The saccular stage results in primitive alveoli. Finally, in the alveolar stage, secondary septa grow into the airspace to increase the surface area of the lung and allow efficient gas exchange through a thin vascularized diffusion membrane (4).

Preterm birth refers to babies born alive before 37 weeks of pregnancy (5). It is estimated that about 7% of pregnancies end prematurely in France (6), 8% in Canada (7) and range between 10 and 14.4% depending on ethnicity in the United States (8). According to gestational age, preterm births are classified into moderate to late preterm (32–37 weeks), very preterm (28–32 weeks), and extremely preterm (<28 weeks). The babies in the last category are the most subject to various complications and have the highest rate of mortality and morbidity.

Respiratory distress syndrome (RDS) (9) is the leading cause of mortality in preterm neonates (10). RDS is the consequence of birth before the emergence of mature lung epithelial type II (PTII) cells that are responsible for surfactant synthesis. The syndrome is characterized by the collapse of airway membranes due to a lack of surfactant. The degree of severity increases with the degree of prematurity (11), morbidity (extremely-low-birth-weight infant) and sex. Indeed, more than four decades ago, Farrell and Avery (9) reported a higher incidence of RDS in male vs. female. This sexual dimorphism has been considered to be caused by the presence of androgens in the male lung that cause a delay in the PTII cell maturation and thus a delay in the surge of surfactant lipid production. For review see (12).

RDS can be an early phase of bronchopulmonary dysplasia (BPD), another lung complication of premature birth. BPD was originally described as a heterogeneous group of lung disorders associated with preterm birth and lung impairment due to mechanical ventilation (13). Today, as the preterm newborn survival rate increases, a new form of BPD has emerged. New BPD is characterized by alveolar simplification as a result of impaired alveolar and capillary development (14, 15). It is a consequence of extreme premature birth with immature lungs rather than a consequence of extended RDS treatment with mechanical ventilation as it was previously described. Its incidence is also higher in males than females (16).

### Steroid Activity: Current Knowledge

During lung development, many regulatory factors exert a negative or positive pressure on the communication between fibroblasts and PTII cells that lead to surfactant production (17). Glucocorticoids have been known to stimulate the production of surfactant-associated protein and stimulate cell maturation through their actions on glucocorticoid receptors in the fetal lung (18). In contrast, androgens lead to a delay in surfactant production and antenatal lung maturation. Indeed, *in vitro* exposure of fibroblasts to androgens decreases their ability to stimulate the maturation of PTII cells (19). As with glucocorticoids, the effect of androgens on the developing lung is mediated by specific androgen receptors (AR) activation (20). Indeed, in TFM (testicular feminization) mouse model, male TFM produce similar surfactant level as female mice (21).

Sex steroids (estrogen and androgen) biosyntheses belong in two pathways. The intra-gonadal endocrine production and the peripheric intracrine production. The endocrine pathway is carried by the ovaries and testes. Active sex steroids are synthesized from cholesterol and released in circulation to have their effects on target tissues. The intracrine pathway depends on intracellular activation of adrenal derived precursors (also named sex steroid precursors, SSP), namely DHEA and androstenedione. These circulating precursors, when presented to tissues with the required enzymes, are converted into active androgens capable of having a direct effect on the cell in the presence of AR (22). Many tissues can exert an intracrine activity. The brain, endometrium, and prostate all have the ability of controlling the occupancy of the AR by conversion of SSPs into active or inactive products. The main enzymes required in androgen metabolism are 17β-hydroxysteroid dehydrogenases (17βHSD) types 5 and 2 which catalyze androgens activation and inactivation, respectively (23).

## Summary

In resume, intracrinology refers to *in situ* synthesis of active steroids in the cell where the steroids have their action (1). Four criteria are necessary to extend intracrinology to the developing lung in context of BPD. First, there must be circulating SSPs in the fetal circulation. Second, the lung cell must be able to transform these into androgens *in situ*. Third, these lung cells must be androgen receptors positive to allow an androgen dependent reaction. Lastly, the androgen action must be part of the lung physiology.

## Summary of data

### Circulating Sex-Specific Steroid Precursors During Pregnancy

The first condition necessary to have an intracrine function is the presence of circulating sex specific steroid precursors (SSPs). The rodent model was considered in the first place to make direct measurements. It is important to note that androstenedione is the main SSPs in rodents whereas in humans DHEA and androstenedione are the main precursors (24). In 1986 Warshaw et al. measured Adione relative levels in rodent (rat) placenta and noted a significant increase in Adione levels on gestational day 18 (25). Accordingly, Hill et al. (26) measured high levels of DHEAS in the umbilical cord and not in the cubital vein of pregnant women. This result strongly suggests that the human placenta is indeed capable of producing sex specific steroid precursors with a peak late in pregnancy. Sex-specific steroid precursors are present in the fetal circulation and they are most likely from a placental origin rather than from the maternal adrenal.

### Androgen Synthesis and Inactivation in the Fetal Lung

17β-hydroxysteroid dehydrogenases (17β-HSDs) (23, 27) 3β-HSDs, (28) and 5α-reductases (29) are the enzymes involved in the synthesis of active androgens from circulating sex-specific steroid precursors (SSPs) in peripheric human tissues. Studies from Milewich et al. (30) confirmed the presence of 3αHSD's and 17βHSD's activities by the capability of the human lung to metabolized SSPs. Following these studies within the developing lung, we aimed to characterize the enzymatic machinery and identify the cells carrying these activities.

### A549 Cells *in vitro*

We first described the ability of pulmonary epithelial type 2 (PTII) cells to synthesize androgens from SSPs *in vitro* using A549 cells. This PTII like cell line was established from a lung adenocarcinoma of an adult male. They have been widely used to study androgen metabolism (31–34). As normal PTII cells, A549 cells can synthesize and secrete surfactant (11) and are androgen receptors (AR) positive (35). Our results show that A549 cells can rapidly convert androstenedione into testosterone with a little formation of 5α-DHT and the inactive 5α-androstane-3α,17β-diol (12). These cells show high levels of expression of 17β-HSD type 5 and 3α-HSD type 3. Overall, A549 cells present a high *in vitro* capacity of synthesizing and maintaining testosterone levels from SSPs while rapidly inactivating DHT (31).

### Lung Fibroblast Cells *in vitro*

We also aimed to describe the enzymatic activity regarding androgen metabolism in perinatal pulmonary fibroblasts using eight normal diploïd cells. Regarding androgen synthesis, none of them showed the capacity to form androgens from SSPs but the opposite reaction was observed. Indeed, the human lung fibroblast cells show a pattern of androgen inactivation where testosterone is mainly converted back into androstenedione and androstanedione without any formation of 5α-DHT. The enzymes responsible for androgen metabolism in lung fibroblasts are 17β-HSD type 2 and 5α-reductase type 1, the latter having a strong substrate preference for androstenedione, the product of 17β-HSD type 2. In addition, all lung fibroblasts studied have the ability to inactivate androgens into their 17-ketosteroid counterparts regardless of sex (36) or fetal age (37).

From this point, we stipulated that androgen could be synthesized by normal male PTII cells *in vitro* but that male and female fibroblasts could modulate the exposure to androgen. To go further into this complex steroid metabolism within the developing lung, *in vivo* experiments were designed.

### Spaciotemporal Regulation of AR and 17β-HSD Type 2-5

17β-HSD type 5 is expressed in PTII-like cell line A-549 (31) in the mouse (38) as well as in fetal human lungs (37). A peak of expression of the enzyme is detected on GD 17.5 in the fetal lung of both male and female mice. This peak corresponds to the surge of surfactant in mature PTII cells (38). Analyses of human lungs from 17 to 40 weeks of pregnancy reveled a similar increase in mid-late gestation (37). These results strongly suggest that both male and female fetal lung can synthesize androgens. In human fetal lungs, 17β-HSD type 5 is located in a small population of epithelial cells in proximal airways and rarely in the distal epithelium where PTII cells are still in development. It is absent from the mesenchyme, smooth muscle cells and endothelial cells (37). These results suggest that 17β-HSD type 5 positive cells are specific to the conducting zone (37).

17β-HSD type 2 is expressed in mice (39) and human fetal (37) lungs epithelial cells and in mesenchymal cells to a lesser extent. This suggests that the androgen inactivation capability is widely distributed in the lung. Around mid-gestation, in mice (37) and humans (39), all epithelial cells from the distal epithelium, most of the proximal epithelium and most mesenchymal cells express the 17β-HSD type 2 genes. Moreover, the expression of 17β-HSD type 2 gene is more intense in the budding portion of developing respiratory ducts as well as in the proximal epithelial cells in early saccular stage. The expression of 17β-HSD type 2 gene also shows a marked decrease between the saccular and alveolar stage (40), but is upregulated from GD 16.5 to 17.5 (38).

In summary, 17β-HSD type 5 participates in the production of androgens while 17β-HSD type 2 modulates the paracrine action of testosterone in lung fibroblasts. Thus, androgen synthesis must be a physiological feature in normal lung development and could play an essential role in cell reprogrammation when the emergence of mature PTII cells occur (38).

### Androgen Receptor Regulation

As the mechanisms of androgen action require the presence of AR, we searched for the distribution of AR within the fetal lung of both male and female.

The AR is expressed at similar levels in male and female mouse with no detectable modulation over time (38). In human, AR is expressed in fetal lungs as soon as 13–16 weeks of pregnancy, mainly in epithelial cells (41) and fibroblasts (42) to a lesser extent. It is detected in the cytoplasm and nucleus of the conducting and respiratory zone cells (37). AR mRNA levels increases between the saccular and alveolar stage of lung development as opposed to 17β-HSD type 2 which decreases (40). These results suggest that the androgen sensibility in the lung begin before the alveolar stage. AR is detected in 17β-HSD type 5 positive and negative cells such as fibroblasts.

In conclusion, both male and female express AR and are capable of synthesizing and inactivating androgen in the period overlapping the surge of surfactant. Thus, the lung is not passively exposed to circulating androgens, it is a dynamic tissue with a fine-tuning action on AR occupancy (11).

### Is the Intracrine System Active?

To make progress, we needed a faster, more precise and reliable morphogenic analysis method. To do so, we developed an automated image analysis program to study the lung development with higher reproducibility, reliability and rapidity than manual analysis. Moreover, a greater portion of the lung and a larger number of samples can be evaluated with this novel method (43).

With this algorithm, we were able to confirm an experimental model of BPD with hyperoxia. Indeed, newborn mice from postnatal (PN) 1 to PN 4 were exposed to 80% oxygen (hyperoxia) and compared with newborn exposed to 21% oxygen (normoxia). The mice exposed to hyperoxia presented drastic changes in density of closed area, a diminution of the relative frequency of closed area under 1,000 mm (2) (alveoli and saccules) and an augmentation of closed area over 1,000 mm (2) compared to mice in normoxia (43). These changes correspond to an alveolar simplification which is the major characteristic of BPD (14, 15).

To evaluate the contribution of androgens during lung maturation, we treated neonate pups with flutamide during the junction between the saccular and the alveolar stage. Flutamide is a pure antiandrogen that binds to the AR but prevents androgens to have an AR mediated response in the cell (44). Both normoxia and hyperoxia flutamide-treated mice showed alveolar impairment. Indeed, at 21% oxygen the relative frequency of closed areas under 1,000 μm (2) decreased and the ones over 2,500 μm (2) increased. The mice exposed to hyperoxia showed the most dramatic alveolar simplification (45). The absence of androgen action does not restore the structure of the lung, this supports a positive role for androgens in lung. As we are in a period of fetal development where the levels of circulating androgen are very low in both sexes but specially in male (46), the androgens in the fetal lung must be formed from SSPs.

If removing androgen response is deleterious to the lung, adding them should reverse this effect. Surprisingly, mice exposed to 80% oxygen then treated with exogenous DHT, had similar morphogenic parameters as mice treated with flutamide alone (45). At 21% oxygen the mice treated with DHT still showed alveolar simplification compared to the control mouse. This result indicates that the complex enzymatic mechanism of androgen activation and inactivation in the lung is active and cannot be bypassed by exogenous androgens in order to exert a physiological action on lung development.

## Conclusion

In 2004, we proposed that androgens should play a positive role in lung development for both sexes (38). Since then, we have accumulated several observations leading to the conclusion that intracrinology concept applies to the developing lung. In order to better understand the action of steroids in the developing lung and demystify the dimorphism associated with the prevalence of BPD in males, a gender-sex-based analysis approach, which takes into account sex, was chosen. This approach has allowed a new understanding of the role of androgens in lung development and has allowed us to demonstrate that the lung is a SEX organ with an INTRACRINE function with therapeutic potential for BPD.

During development, the lungs and particularly the female lungs are not exposed to a significant amount of circulating androgens (47). Nevertheless, the androgen receptor has been found in the nucleus of several cells in developing lungs in both sexes (38). Moreover, the fetal lung in both sexes is capable of synthesizing testosterone from sex-specific circulating precursors (31) through 17β-HSD type 5 expressed in PTII cells (37). On the other hand, fibroblasts expressing 17β-HSD type 2 (37) inactivates androgens (36). Finally, removing all androgen response in the cells with flutamide causes alveolar simplification (45), and the alveolar impairment cannot be reversed with the administration of exogenous DHT, because the specific enzymatic machinery has been bypassed.

## Data Availability Statement

The raw data supporting the conclusions of this article will be made available by the authors, without undue reservation.

## Ethics Statement

The animal study was reviewed and approved by Laval University Animal Care Committee.

## Author Contributions

YT heads the laboratory. He has supervised the research work of the laboratory for the last 30 years. He supervised AM-L in the writing of this chapter. Both authors contributed to the article and approved the submitted version.

## Conflict of Interest

The authors declare that the research was conducted in the absence of any commercial or financial relationships that could be construed as a potential conflict of interest.
